# How do healthcare consumers process and evaluate comparative healthcare information? A qualitative study using cognitive interviews

**DOI:** 10.1186/1471-2458-9-423

**Published:** 2009-11-20

**Authors:** Olga C Damman, Michelle Hendriks, Jany Rademakers, Diana MJ Delnoij, Peter P Groenewegen

**Affiliations:** 1 NIVEL (Netherlands Institute for Health Services Research), PO Box 1568, 3500 BN, Utrecht, the Netherlands; 2 Centre for Consumer Experience in Healthcare, PO Box 1568, 3500 BN, Utrecht, the Netherlands; 3 TRANZO (Scientific Centre for Transformation in Care and Welfare), Faculty of Social and Behavioral Sciences, Tilburg University, PO Box 90153, 5000 LE, Tilburg, the Netherlands; 4 Department of Human Geography, Department of Sociology, Utrecht University, PO Box 90115, 3508 TC, Utrecht, the Netherlands

## Abstract

**Background:**

To date, online public healthcare reports have not been effectively used by consumers. Therefore, we qualitatively examined how healthcare consumers process and evaluate comparative healthcare information on the Internet.

**Methods:**

Using semi-structured cognitive interviews, interviewees (n = 20) were asked to think aloud and answer questions, as they were prompted with three Dutch web pages providing comparative healthcare information.

**Results:**

We identified twelve themes from consumers' thoughts and evaluations. These themes were categorized under four important areas of interest: (1) a response to the design; (2) a response to the information content; (3) the use of the information, and (4) the purpose of the information.

**Conclusion:**

Several barriers to an effective use of comparative healthcare information were identified, such as too much information and the ambiguity of terms presented on websites. Particularly important for future research is the question of how comparative healthcare information can be integrated with alternative information, such as patient reviews on the Internet. Furthermore, the readability of quality of care concepts is an issue that needs further attention, both from websites and communication experts.

## Background

Following the increased emphasis on transparency and consumer choice in healthcare, much effort has been made to publicly report healthcare performance. The aim is to stimulate informed decision making in healthcare and ultimately to improve healthcare quality. Therefore, comparative healthcare information should be effectively adopted and used by healthcare consumers. There is some evidence that people, particularly unsatisfied or new consumers on the healthcare market, are interested in the information [1-4]. Nevertheless, several studies have shown that publishing information on healthcare performance has had little impact on consumers' use of it [5-7]. One of the explanations for this lack of use considers that online performance information may be poorly constructed and unadjusted to human information processing strategies [8-10].

Despite research evidence and conventional wisdom that comparative healthcare information is complex and human processing capacities are limited [11-13], providing healthcare consumers with large amounts of (mostly online) public healthcare reports has continued. If we want this kind of information to be more effectively used by consumers, it is necessary that they can easily process the information[14]. Although there has been research on how healthcare consumers evaluate and use health-related websites, [15,16] the specific bottlenecks that consumers face when processing comparative healthcare information have not been thoroughly examined. In addition to studies on design features [17,18] and website usability, an in-depth understanding of how consumers manage comparative healthcare information is thus needed.

### Information processing

From cognitive science and decision making literature, we know how information can be processed by consumers. Broadly, people either think in an analytical (rule-based) or experiential mode [19]. The analytical mode concerns conscious, deliberative, attribute-by-attribute reasoning, which is relatively slow. Dijksterhuis and colleagues [13] argued that human consciousness has limited capacity; causing consumers to take into account only a subset of relevant information. Therefore, the analytical mode is usually applied when information is relatively simple. In contrast, the experiential mode consists of more associative, automatic reasoning, occurring relatively fast. People often apply this processing mode, using shortcuts or intuitive heuristics, especially when large amounts of information are concerned [20-22]. Usually, consumers only scan information [23], looking for information they want [24], and in the light of questions they already have in mind, their knowledge, and their expectations [25,26].

When it comes to making decisions, several 'decision strategies' (that is, methods whereby decision makers search through the decision problem) have been described in the literature [21,22]. Generally, a decision strategy contains a search for the relative importance of attributes, and a specification of cutoff values and preferences across attribute levels. The most common strategies are shortly described in Table 1. Decision strategies are often used in combination, for example eliminating poor alternatives in an initial phase, and examining remaining alternatives in more detail in a second phase [21].

**Table 1:** Overview of common decision strategies*

Decision strategy	Short description
Weighted addititive (WADD)	Taking into account the values of each alternative on all relevant attributes; considering the relative importance of each attribute; multipying weights times attribute values; summing weighted attribute values over all attributes.

Additive difference (ADDIF)	Comparing pairs of alternatives directly on each dimension; determining the differences between subjective values of alternatives on a particular dimension; applying weighting function to each difference and summing results over all dimensions to obtain overall relative evaluation of two alternatives.

Equal weight (EQW)	Choosing on basis of the sum of all values; ignoring information about relative importance.

Elimination-by-aspects (EBA)	Assessing most important attribute; eliminating all options that are not satisfactory with respect to that attribute; repeating for next most important attribute and so on, until there is one option left.

Satisfying (SAT)	Choosing the first option that is satisfactory.

Lexicographic (LEX)	Assessing most important attribute; selecting the option that has the best value on that attribute.

Lexicographic semiorder (LEXSEMI)	Assessing most important attribute; selecting the option that has the best value on that attribute; including notion of selecting alternatives that are within just-noticeable difference (JND) of the best alternative.

Majority of confirming dimensions (MCD)	Choosing by comparing pairs of alternatives; winner is compared with the next alternative in the set; simplified version of the ADDIF strategy (only direction of differences is considered, not the magnitude).

Frequency knowlegde (FRQ)	Counting the number of good and bad features; the option with the smallest numer of bad features or the option with the biggest number of good features is chosen.

Habitual heuristic	Choosing what you chose last time.

Affect referral	Recalling from memory previously formed evaluations for familiar alternatives; choosing acoordingly.

Price-oriented	Buying the cheapest product.

In store	Buying the first product you find.

* The decision strategies are based on descriptions in Payne, Bettman, and Johnson (1993)^21 ^and Devetag (1999).^22^

Choices based on comparative healthcare information typically involve the following demands: 1) processing technical terms and complex ideas; 2) comparing multiple alternatives on several attributes; and 3) weighting various factors according to individual preferences [27]. These processes and trade-offs are known to be difficult [28] and provoke fast and frugal decision making [29]. Furthermore, comparative healthcare information seems to produce preferences that are 'constructed' while sorting through information ('constructed preference') [30,31]. This means that consumers have no fixed ideas about their priorities in healthcare quality, and construct them depending on how the information is provided.

To summarize, it is known which general processing strategies can be applied by consumers, but relatively little is known with respect to comparative healthcare information. The literature suggests that it is a complex job to process comparative healthcare information, and Internet research has identified many guidelines to improve website usability. However, hardly any studies have comprehensively examined the information processing strategies of consumers themselves. To be able to understand the difficulties and bottlenecks consumers face, an open, qualitative approach using real online information is therefore needed. With this study, we aimed to gain insight into consumers' own thoughts, interpretations, and evaluations of this kind of information. Our research question was: *"How do consumers process and evaluate comparative healthcare information?'*"

## Methods

### Cognitive interviews

A descriptive qualitative approach was adopted to explore consumers' thoughts about and interpretations of comparative healthcare information. We chose to investigate the topic qualitatively to be able to understand the experiences of consumers themselves and to investigate the relevant themes in-depth. We performed semi-structured cognitive interviews with consumers, who were prompted with existing Dutch comparative healthcare information on a computer screen. Cognitive interviewing is a technique for investigating thought processes people use as they sort through information and make decisions [32]. To gather rich and detailed information, participants were instructed to think aloud while they viewed the information. Furthermore, we posed open-ended questions about the material using a topic list with standardized themes. Table 2 summarizes the content of the interview protocol. Participants were allowed to go through information and surf to web pages behind the initial page.

**Table 2:** Summary of interview protocol

Part of the interview	Key text/questions
Introduction	Today I will show you information about the quality of healthcare on the internet. We would like to hear your reaction to the information.

	The purpose of the interview is to let you 'think aloud'. You are encouraged to say anything that comes into your mind. We are interested in all your reactions.

	Are there any questions before we start?

Part 1: Thinking aloud	Can you tell me what you are thinking as you see this information?

	Can you tell me what this information is about?

Part 2: Probing	According to you, what is the purpose of this information?

	What do the presented stars mean to you?

	Can you explain the term "personal communication of employees" in your own words?

	Why do you think that the aspect "public avalibility of data" is presented to consumers?

Part 3: Choice task	If you would choose a hospital/health plan based on this information (for yourself or for someone close to you), which hospital/health plan would you choose?

	If you would choose a hospital/health plan based on this information (for yourself or for someone close to you), what would this information mean to you?

Conclusion	Are there any further questions or things you would like to say?

We performed the interviews in a small, private room, and an assistant made notes during the interview. Interviews were recorded on audio tape with permission of the interviewees. Participants filled out an informed consent form and a questionnaire about demographic variables. After that, they were rewarded with a token gift, namely a gift voucher of 15 euro. Each interview lasted about one hour. The interviews were performed by a team of five researchers, who had a joint instruction before the start of the interviews. After a first round of interviews, interviewers were debriefed on the main findings and aspects to pay attention to in the next set of interviews.

### Materials

**Figure 1:**
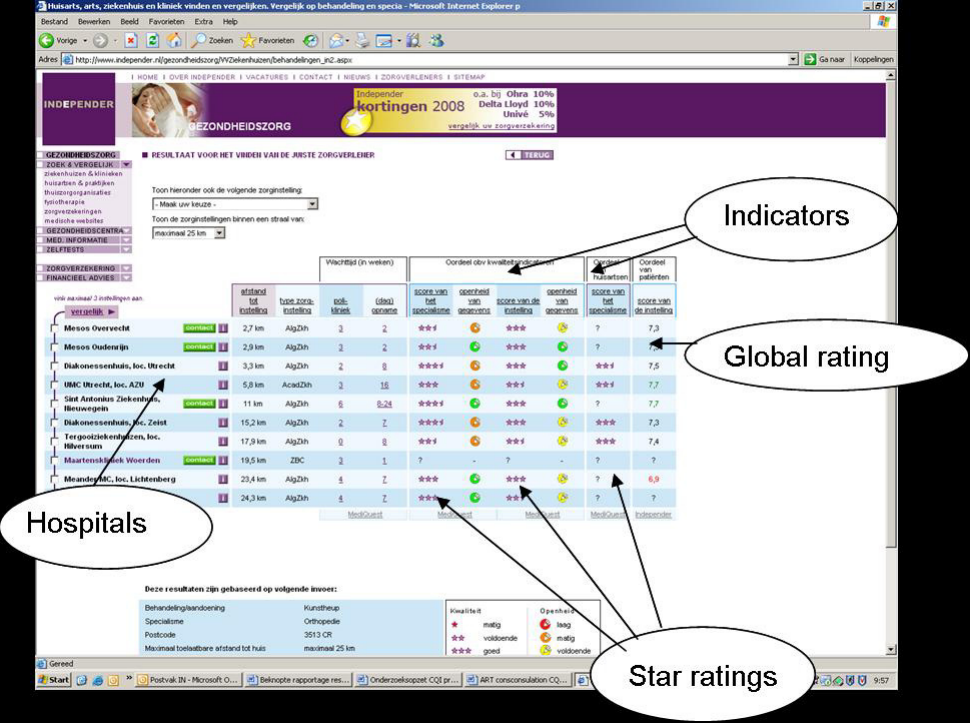
**Comparative information on hospital quality concerning hip surgery http://www.independer.nl**. The following indicators are shown in this information: 'distance to the hospital', different types of waiting times (numbers), 'quality indicators' (stars and colored icons), 'opinion of family doctors' (stars), and 'opinion of ex-patients' (global ratings). The 'quality indicators' stem -for the most part- from objective performance indicators collected by the Healthcare Inspectorate. The data of 'opinion of family doctors' and 'opinion of ex-patients' are generated by (online) surveys among family doctors and patients.

**Figure 2:**
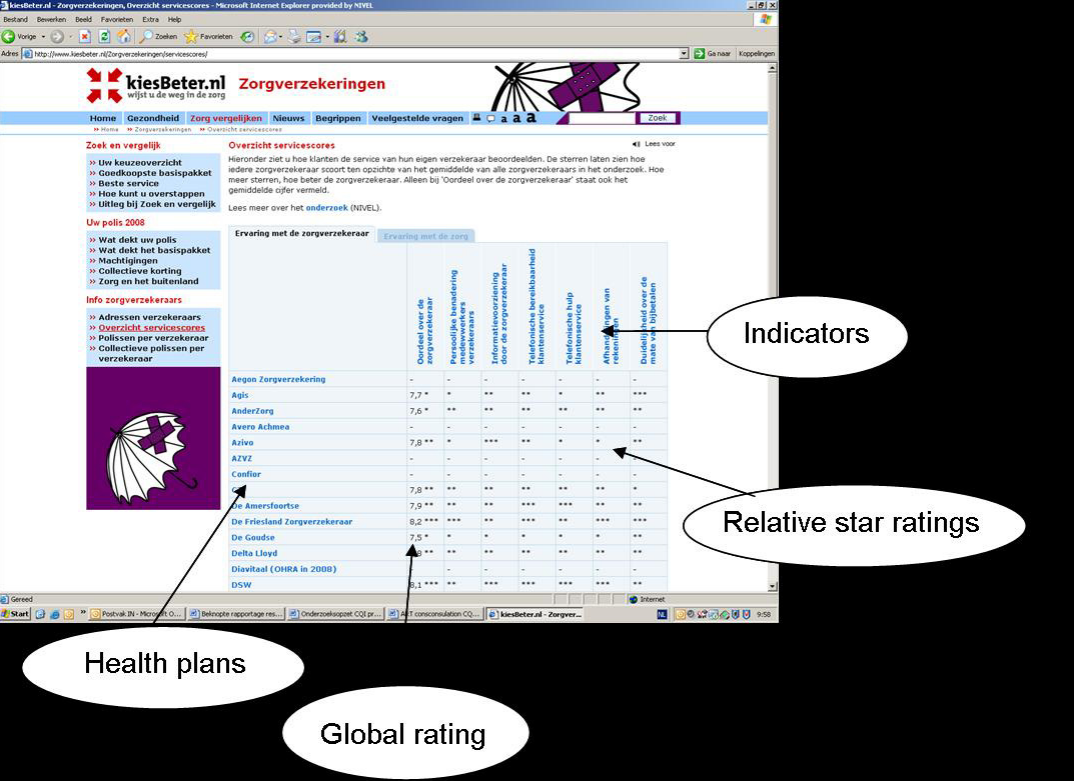
**Comparative information on quality of health plans http://www.kiesBeter.nl**. The indicators consist of a global rating (rating and stars) and different quality themes (stars). The data stem from a survey among health plans'enrollees (about their experiences with their health insurer and the received healthcare).

**Figure 3:**
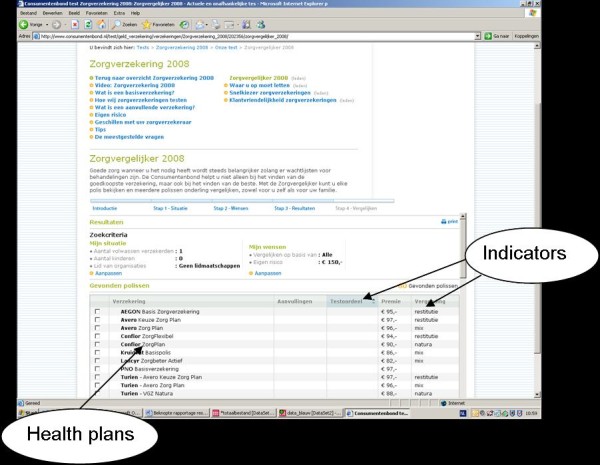
**Comparative information on quality and premium of health plans http://www.consumentenbond.nl**. The indicators shown are: 'test opinion', 'premium', and 'reimbursement'. The data are generated from health insurers and from research using surveys among health plans'enrollees (about their experiences with their health insurer and the received healthcare).

Participants were provided with three Dutch web pages containing comparative healthcare information as visual prompts: 1) information on the quality of hospital care concerning hip surgery (Figure 1[33]); (2) information on the quality of health plans (Figure 2[34]); and 3) information on both quality and premiums of health plans (Figure 3[35]). At the time of the interviews, these websites were relatively well-known public reporting initiatives in the Netherlands. In addition, we chose to test these websites because clinical performance indicators -defined by the Dutch Inspectorate for healthcare [36] are presented- as well as patient experience information measured with the Consumer Quality Index (a set of standardized patient surveys) [37]. The pages were presented in six different orders (3*2) to control for potential order effects.

### Data collection and sample

We invited a sample of 157 members of a Dutch health plan enrollees panel (*VGZ Insurants Panel*) to participate. The aim of this panel is to gather information on consumers' experiences with and expectations of healthcare in general and their health insurer in particular. Panel members were previously recruited through an announcement in the magazine of health insurer VGZ and are all enrollees of this health insurer. To guarantee panel members' privacy, the panel is managed by the NIVEL; the health insurer is ignorant about who of their enrollees are panel members. The panel is registered by the Dutch Data Protection Authority (no. 1309664). Approval by an ethics committee is not necessary under Dutch law. The 157 panel members were selected by the researchers based on travelling time to the interview location (maximum of 45 minutes) and age (maximum of 85 years). The selected individuals received an invitation letter from the researchers to participate in the present study. In total, 22 consumers (14%) responded, of which 20 actually participated. Table 3 shows participants' characteristics.

**Table 3:** Participants' characteristics

Variable	N	%
*Age*		
18-34	1	5.3
35-54	4	21.1
55-64	7	36.8
65-74	6	31.6
>74	1	5.3

*General health status*		
Excellent	3	15.0
Very good	4	20.0
Good	10	50.0
Fair	2	10.0
Poor	1	5.0

*Gender*		
Female	9	45.0
Male	11	55.0

*Education*		
Low (primary education)	0	0.0
Average (secondary education)	9	45.0
High (tertiary education)	11	55.0

*Ever visited http://www.kiesBeter.nl?*		
Yes	4	20.0
No	16	80.0

*Ever visited http://www.independer.nl?*		
Yes	4	20.0
No	16	80.0

*Ever visited http://www.consumentenbond.nl?*		
Yes	8	40.0
No	12	60.0

### Analysis

The original audio tapes were transcribed and the transcriptions were analyzed by one researcher. A second researcher independently analyzed a subset of the transcriptions. Both researchers conducted descriptive thematic analysis, consisting of an open coding and an axial coding phase [38,39]. Open coding was characterized by fragmenting [40]: relevant themes were extracted, categorized and classified. After the research team verified the themes, relationships between categories were identified in the axial coding phase. Since we were most interested in consumers' own spontaneous interpretations and information processing, we focused on data derived from thinking aloud. Subsequently, we analyzed answers to specific questions. In the descriptions of the emerging themes, we used the following guideline to connote the quantity of how often themes were mentioned [41]. *Many*, *often, frequently *and *generally *are used when a theme is mentioned by more than 75% of participants; *common *and *several *when mentioned by about 50% to 75% of participants; *some *when mentioned by about 25% to 50% of participants; *few *when mentioned by less than 25% of participants.

## Results

Data analyses resulted in the identification of twelve themes, which are described in this section and illustrated by interviewees' quotes. All quotes were translated from Dutch by the first author, and checked by the second author. We categorized the themes according to the main areas of interest in the study: a response to the design and content of the information (thinking aloud), the purpose of the information (probing), and the use of the information (choice task).

### Response to the design

Participants often spoke about the design of the website, focusing on aspects such as the amount of information on one webpage (theme 1), information complexity and organization (theme 2), usability of the webpage (theme 3) and the appearance of the information (theme 4). Participants wanted to go quickly through the information and preferred information that is clear at first sight. Generally, negative comments were made about the amount of information (theme 1), such as the following:

How I feel about it? It is too much. I have to consider it line by line. It's too much for one webpage.

The number of health plans is overwhelming. You should view all and then wonder 'what was at the top?'. So you must actually move back and forth. I would not prefer this.

Well, I have to go through a lot, based on this information. Because if you have a number of your own criteria, you still got to do a lot of work to specifically find out.

It is clear that participants felt overwhelmed by the amount of information, which sometimes caused them to stop considering it. It was striking how often consumers said that it was too much immediately after providing them with the information. Some people described their feeling by words as 'overwhelming', 'confused', and 'disorderly'. In contrast, some participants were satisfied with the presented quantity.

Comments were made about the complexity and organization of the design (theme 2):

Well, I think that this website appears calm, compared to the other one. It is more conveniently arranged, and has clear components. This really works for me.

I mean the structure of the information. I feel that the structure is not straightforward. But that's also a personal matter, I think.

From these quotes we see that interviewees' evaluation of the complexity was related to how the information was organized. Interviewees also frequently touched upon the usability or user-friendliness of the website (theme 3):

I have to read the information vertical. That's very bad, because I have to turn my head.

It is not clear that these aspects are clickable.

These quotes tell us that the vertical display of quality indicators and the clickability of aspects of choice are barriers to an easy use of the information. Other barriers were mentioned as well, namely the absence of an option to fasten text in the upper part of a table while scrolling down, and the ambiguous display and content of mouse-overs.

Concerning the appearance of the information (theme 4), interviewees criticized the layout, type face, and the use of colors, as the following quotes illustrate:

I think it is just a messy layout. Letters that don't fit in a box. It is a noisy site, Look, holes and corners are everywhere.

This site is nice and open. A lot of white and bright colors. And a large font.

### Response to the content of information

Participants' thoughts focused on different aspects related to the actual information content: the importance of quality indicators (theme 5), the interpretation of information (theme 6), a comparison of the information to their own experiences and ideas (theme 7), and the quality of the presented information (theme 8).

Many interviewees spontaneously attributed importance to the presented quality indicators (theme 5), and further focused on those aspects that they prioritized:

I focus on the opinion of family doctors. That's something that I find important. What my family doctor would think about the quality of hospitals.

Let's see: I think waiting times are important. I see that hospital A has waiting time period of 7 weeks, which I think is just too long.

Almost all information was considered important, and some interviewees even wanted more detailed information, which is hard to reconcile with their feelings of being overwhelmed by the amount of information. The following quote nicely illustrates this inconsistency:

I'd like to have more background information. What's the meaning of the stars? How much stars are there? What's the purpose of "performance indicators"? The number of beds? The number of single and double rooms? That can be included in the information. But it has to be more straightforward than it is now.

Most participants tried to interpret the information, and misunderstood a lot of it (theme 6):

"Opinion of ex-patients" means that these patients had a new hip and evaluate whether they are satisfied about it. Perhaps these patients had to fill out a questionnaire about that. But whether these questionnaires are used for this website. I don't know. Perhaps through the Internet. But it contains an opinion on satisfaction, I guess.

The bar chart says nothing. It is not clear what this actually means, "reimbursement of claims". Then you get scores of never, sometimes, usually, and always. Does this mean that a claim is always reimbursed in one year?

The second quote illustrates that bar charts were incorrectly interpreted. The scores of never, sometimes, usually, and always actually indicate how often claims were reimbursed correctly. Similarly, interviewees had difficulty interpreting symbols, especially when these were based on relative performance scores (performance that is worse than average, average, or better than average). The association between relative and absolute performance was not always clear, as can be seen in the following quote:

Health plan A has one star on all aspects. That's very bad. In my mind, they shouldn't have given one star to a rating of 7.4. That is too high. So, I doubt whether this rating of 7.4 really is an answer of respondents. I don't believe that.

This participant had trouble to understand that one star means 'a worse than average performance', and not an absolute 'bad performance'. Presenting absolute global ratings simultaneously (7.4) caused confusion.

Furthermore, participants found it hard to understand conflicting information when, for example, some hospitals performed good on one quality aspect, but bad on another aspect. One participant stated,

But that's very strange. Look at this. Here we find a contradiction. Look, that can't be possible. The performance of this hospital according to patients is very high. But the "public availability of data" is not so well. Oh, but wait a minute. Oh, I see. If you look at it a little bit longer, all sorts of questions come up. But now I think I understand. Hospital C is very reserved as to providing quality information. Oh dear, I find this very annoying. If I'd only had a fast glance, I wouldn't have understood.

The naming of several quality indicators was poorly interpreted, such as 'reimbursement', 'restitution', 'test opinion', 'public availability of data', 'quality indicators', and 'clinical specialty'. For example,

I don't understand the term "reimbursement". Perhaps I can read somewhere what that means? "Restitution", or "mixed", or "in kind". Does "reimbursement" mean that I get my medication directly?

"Quality indicators" represent the extent to which they pay attention to the patient. That's interesting, of course. Because it indicates whether they find patients important. Well, not always, but more and more, though.

In reality, the term 'reimbursement' refers to how insurance claims are processed: either directly, without interposition of the consumer, or indirectly via the consumer. 'Quality indicators' do in reality reflect objective performance indicators, and not merely patient-centeredness. Global ratings were often misinterpreted as well. Many participants thought that global ratings were composed of other presented indicators. In fact, global ratings are given by patients on a scale from zero to ten on a questionnaire item. In contrast, some terms were well understood, such as 'opinion of ex-patients', 'information', and 'telephone assistance'.

We further learned that many participants were comparing the presented information to their own experiences and ideas (theme 7):

Overall I think the score of health plan A is a bit low. My experience is that they are not that bad.

But Hospital C is my first choice, although I live in place A. I just don't like the two hospitals near place A.

We observed this tendency in interviewees' responses to the information content, but it appeared to be related to their hypothetical choices as well:

I don't want to go to hospital B because of an old-fashioned idea that I have. Because there were several incidents in my surrounding in that hospital. And that's why I'm not inclined to go to that hospital, as good as it may be now.

This quote tells us that the interviewee would not choose for hospital B, because its performance conflicted with ideas already in mind.

A final aspect related to participants' reactions to information content was that the quality of the information itself was frequently questioned (theme 8):

The "opinion of ex-patients". Well, maybe only two patients were questioned? So I'd like to know more about this website. I'd like to know how the opinion of ex-patients, how that works. Was the sample large enough?

When there are question marks, just like here, you can question the adequacy of the information.

From these quotes, we see that questions were raised about the completeness and reliability of the information. In addition to these issues, interviewees also commented on the magnitude of quality differences.

### Use of information

Participants' thoughts often focused on the potential use of the information in daily life (theme 9):

I'd never make a decision based on this kind of information. Perhaps rather on personal experiences of others, and I would ask others.

I didn't know that this kind of information is available. So, now that I know, I think it's interesting information. It's tempting to look at it at some time. So I think I would look at it.

If I had to make a choice, I would look for things that I find important. But I think I know to which hospital I'd want to go. That's because I have experience with that hospital and I'm satisfied. If you are very satisfied with a particular hospital, and that hospital does not have so many stars, I'd rely on my own experience.

These quotes illustrate the variation among consumers' interest to use the information in daily life. Some interviewees thought that comparing providers on different quality aspects is a tough and time consuming activity. Others felt that information could be a helpful tool for their healthcare decisions. One agreed that other information sources were required to make an informed choice, either instead of or complementary to comparative information. Frequently cited information sources were their own experiences and perceptions, experiences of relevant others, provider image, advice of family doctor and health insurer, and media reports. How the information could be used in daily life (theme 9) appeared to be associated with the design and content of the website, such as the amount of information, and with the perceived relevance of quality indicators.

Interviewees also differed concerning the decision strategies used to make a hypothetical decision during the choice task of the interview (theme 10):

Well, I find quality of care most important. Yes, the score of "clinical specialty". And then I'd choose for Hospital A. Because that hospital is the nearest. And because Hospital A still has a good reputation. That reputation is not contradicted on this website. But, apparently, 37% of the requested data were provided. I'm not immediately sad with a performance of three stars on "clinical specialty". And the "opinion of patients". I think that's important, but they do not highly differ from each other, I see. And besides, this score is all right for hospital A, a score of 7.7.

If I have to choose now, on basis of these data, I would find it hard and complicated. Perhaps then I'd focus on, God help me, the global rating of 8.2.

I concentrate on aspects where large differences exist. These are found on "clinical specialty". That's where differences exist. "Opinion of family doctor" is not available. But especially this one with two stars. I think that's bad, compared to the others.

Many of the strategies listed in Table 1 were used. Several participants systematically weighted the information. For example, they examined quality aspects one by one (WADD) or first defined most important aspects and then compared performance (LEX). Additionally, strategies by which providers were excluded one by one when performance did not meet requirements were often used (EBA). Frequently observed as well was the strategy to count up the number of good and bad scores on different aspects (FRQ). However, more simple associative strategies and shortcuts were also used. Some interviewees, for instance, chose the provider with the highest global rating (Performance Oriented). Yet, even more simple strategies, such as choosing the provider first named (In Store), providers with a familiar name (Affect Referral), providers chosen before (Habitual Heuristic), or the cheapest provider (Price Oriented), were used. Most participants adopted a mix of the above mentioned decision strategies, particularly those who systematically weighted information.

Many interviewees had difficulty making the hypothetical decision. First, several participants were not able to complete this task, because they needed additional information from other sources. Second, it took most consumers lots of time to complete the task. Third, several participants used shortcuts to decide, which indicates that the amount of information was too large for them to process systematically. Apart from these difficulties, we found incongruity between what consumers said to find important or what they would do, and what they actually did when making a choice. For example, during thinking aloud, several participants came up with aspects that they prioritized. Later on, however, these aspects were not weighted in their decisions.

### Purpose of the information

Participants had clear ideas about the direct purpose of the information (theme 11). Although a few consumers thought that the information was designed to inform health insurance companies or hospitals themselves, most participants related the information to consumer choice in healthcare:

This information attempts to rate hip surgery quality. The aim is to get some insight into this quality. Then I can choose what's important to me. Should the clinician be excellent? Should the hospital be near? You get some information on these aspects.

The intention is to provide a summary of all options, so we can make choices in healthcare and live happily ever after.

If people want more freedom of choice in their health insurance, they obviously want to know what they can ensure, what is available, how fast and reliable such insurers are. That's what you are looking for when using this information.

We see that consumers generally knew why the information is presented to them. However, this does not necessarily mean that they actually wanted to use it, for example, if there are few provider-differences.

Participants' thoughts also concentrated on the purpose of different quality indicators (theme 12). Most consumers were able to describe the purpose of different quality indicators, in particular when they saw benefits of presenting the information:

"Opinion of family doctor". Family doctors do have an idea about how clinicians do their work. And these doctors give their view as well. They give stars, or they say they have no idea.

The global rating for health plans is presented because people are used to think in numerals. Therefore, a rating from 0 to 10 immediately says something. If a health plan has a global rating of 5, everyone thinks 'Oh no, that's not where you'd have your insurance'. It's as simple as that.

## Discussion

We described how consumers process and evaluate comparative healthcare information. People applied various strategies to process the information they were provided with, especially when making hypothetical decisions. In line with the findings of Harris [2], variation was shown concerning consumers' willingness to use the information. Nevertheless, we detected a main line from consumers' thoughts, classified into twelve themes. These themes were categorized under four important areas of interest: (1) a response to the design; (2) a response to the information content, (3) the use of information, and (4) the purpose of the information.

### Study strengths and limitations

Our study is the first to investigate in-depth consumers' own thoughts about Dutch comparative healthcare information. An important strength is that real online information was used, with all its complexities included. We used three different websites which are typical for websites internationally [42], and the results were of the same order for these three websites. The open qualitative approach resulted in detailed information about the interpretations and experiences of consumers themselves. Our findings therefore provide a thorough and valid understanding of consumers' experiences and the difficulties that they face. However, our small scale study does not allow for specific recommendations concerning presentation formats. More controlled experiments and observational studies are needed to further investigate decision making using online comparative healthcare information.

A limitation of our study is that neither low educated people nor ethnic minorities participated, although they were invited. This might suggest that certain consumer subgroups are not interested in comparative healthcare information, think that participating is too difficult, or that their jobs or lives are less flexible. Lower educated people are known to have more difficulty understanding healthcare quality information. In addition, the use of Internet is limited among lower educated people and ethnic minorities [43]. This means that their use of the information might even be more complicated than was shown among our participants. Further research should be conducted to investigate these potential problems concerning accessibility of information and equity.

Our findings were also limited by the fact that our participants were not facing a real decision. We forced consumers to choose, which can bias the results towards the 'safer', more average option [44]. Patients facing a real decision in healthcare might weigh other aspects than volunteers in hypothetical choices. Real healthcare consumers usually do not have a 'no choice' option either, though they can decide to leave the choice of a provider to their family doctor who refers them, or-in market research terms- who acts as a 'surrogate consumer' [45]. Is it important to realize that real decisions in healthcare involve many factors within a healthcare trajectory, rather than merely visiting one website to get informed [46].

### Important findings

A key finding is the tension between the great amount of information consumers stated to find important and how sporadically they actually incorporated this information into their decisions. Furthermore, ideas on which quality aspects are important to consider changed during the course of the interview. This inconsistency between (initial) interest in certain information and (later) leaving out of consideration has been found previously [15,16,28]. It suggests that preferences are constructed gradually during the interview [16,30,47], and are not as predictable as is sometimes assumed. As already mentioned as a study limitation, the prescriptive nature of our question (*what would you do.?) *might contribute to differences in what people said to what they actually did. Another explanation might be found in the data itself; when there are few provider-differences on aspects that one considers important, that aspect is not weighted in the eventual choice, though it is still considered important.

Considering the difficulties that participants experienced when processing the amount of presented information and making a choice, we want to emphasize the perceived barrier of too much information. It is known that people can only process about six pieces of information at a time and are easily overwhelmed by information [11]. Therefore, providing all available information is not the most effective way to stimulate informed choices [48,49]. As argued by Eysenbach, websites do not always need to be complete and present the full information spectrum about a particular disease or healthcare topic. Indeed, consumers are able to gather information from various sources and sites [16,50]. Therefore, websites should rather provide conceivable overviews with small numbers of providers and the most relevant quality aspects, and offer more detailed information into step-by-step pages, an approach corresponding to humans' need for generic to specific information [14,51]. This deep-linking approach, which has been frequently cited in the broader context of consumer health informatics, [16,23] could reconcile consumers' desire for more information without overwhelming them. Gerteis and colleagues [52] suggested to use evaluative formats (for example stars) on a first page and let consumers drill down to more detailed bar graphs.

Consumers found it hard to process contradictory information, such as a hospital with high performance on one quality aspect, and low on another aspect, which also corresponds to previous findings [28]. Conflicting information asks for more cognitive effort, which forces consumers to make trade-offs of important aspects and to rely on intuitive heuristics. Comparative healthcare information usually contains contradictory information. Initiatives to prepare or train consumers about potential contradictions might remove some confusion. However, effectively processing contradictory information requires relatively complex strategies and will continue to be difficult.

Only a few consumers deliberately processed all information. More often, only parts of the information were considered, particularly information about familiar providers. This suggests that consumers are not interested in all information, but rather want to check how particular providers perform compared to others. This corresponds to what we know from cognitive science about interpretation in light of questions and information already in mind when viewing information, such as reviews of other patients or media reports [16,26]. Therefore, it seems important to relate comparative healthcare information to alternative information that consumers find familiar [48]. For example, anecdotal or patient review information (such as on *NHS Choices *in England [53] and *Consument en de Zorg *in the Netherlands [54]) might be an interesting source of additional consumer information. Further research is needed to assess whether and how these different types of information should be integrated.

Various strategies were applied to choose providers, varying from systematic reasoning to more intuitive, experiential reasoning using only parts of information. Both alternative-based reasoning and attribute-based reasoning were used, which are both known to be used when information is presented in a matrix format [55,56]. In terms of web design, it means that pages presenting information need to be highly flexible, and preferably allow selections on both prioritized aspects and particular providers of interest.

A substantial number of the participants was interested in the presented information, and understood the purpose of the information. In line with a previous qualitative study [57], consumers appear to comprehend information among main lines, but have difficulty understanding more detailed information and concepts. Findings seem to contradict the notion of some researchers that consumers are not interested in comparative healthcare information. Perhaps the healthcare market is different from other markets where people prefer not to choose, e.g. the energy market [58,59], in the sense that healthcare is a product that is of interest to people. There are many documentaries and talk shows about health and healthcare, and hardly any about gas and electricity. So even if consumers are not willing to choose, they can still find healthcare information interesting.

## Conclusion

Although it is not possible to generalize our findings or to create specific guidelines, some general conclusions can be made. We identified several barriers that consumers face when processing comparative healthcare information; in particular the information amount and the interpretation of detailed information. In addition, several interviewees could not let go of factors outside the task, and many struggled with the choice task. Many of the themes derived from the interviews and subsequent conclusions correspond to existing knowledge from cognitive science and Internet research. In other words, what is generally known about good website design and usability also applies to online comparative healthcare information. For example, clear overviews and flexible navigation options are important conditions for an effective use. Two topics that more specifically concern comparative healthcare information need further attention:

(1) First, the presentation of comparative information in relation to alternative information from other sources. Access to anecdotal or patient review information could make the comparative information -being more factual and less animated- more relevant and easier to process. However, such initiatives are likely to increase the amount of information. In our opinion, only the quality themes that contribute to informed decisions should be presented. Future studies should test such minimum sets of comparative information in combination with alternative information.

(2) Second, the readability of the information in terms of specific quality themes and the overall concept of healthcare quality. Although numerous studies have recommended easy reading text, our study shows that concepts and text about comparative healthcare information are still not comprehensible. Any website presenting comparative healthcare information should test the specific naming of quality themes, preferably using cognitive interviewing techniques. In addition, we should use the experience of communication experts when it comes to communicating the quality of care concept.

In light of more general experiences of consumer choice stress, the results are relevant for future expectations of consumer choice in healthcare. Prospects about consumers' own active use of online comparative healthcare information as a stimulus for high quality healthcare may have to be tempered, [60,61] at least until more effective presentation has been demonstrated. Given that comparative information will continue to be difficult, especially for consumers having low health literacy, public health policy could search for alternative pathways to get healthcare consumers informed about healthcare quality.

## Competing interests

The authors declare that they have no competing interests.

## Auhors' contributions

OD participated in the design of the study, performed the data collection and interviews and analyzed the results. Furthermore, she wrote the draft version and revisions of the manuscript. MH also participated in the study design and performed interviews, and she was involved in drafting and rewriting the manuscript. OD and MH together interpreted the results. JR participated in the design and coordination of the study, and helped to draft the manuscript. DD and PP conceived of the study and helped to draft and rewrite the manuscript. All authors read and approved the final version of the manuscript.

## Pre-publication history

The pre-publication history for this paper can be accessed here:

http://www.biomedcentral.com/1471-2458/9/423/prepub
